# A simple psychophysical procedure separates representational and noise components in impairments of speech prosody perception after right-hemisphere stroke

**DOI:** 10.1038/s41598-024-64295-y

**Published:** 2024-07-02

**Authors:** Aynaz Adl Zarrabi, Mélissa Jeulin, Pauline Bardet, Pauline Commère, Lionel Naccache, Jean-Julien Aucouturier, Emmanuel Ponsot, Marie Villain

**Affiliations:** 1 Université de Franche-Comté, SUPMICROTECH, CNRS, Institut FEMTO-ST, 25000 Besançon, France; 2 Department of Physical Medicine & Rehabilitation, APHP/Hôpital Pitié-Salpêtrière, 75013 Paris, France; 3 Paris Brain Institute (ICM), Inserm, CNRS, PICNIC-Lab, 75013 Paris, France; 4 Science & Technology of Music and Sound, IRCAM/CNRS/Sorbonne Université, 75004 Paris, France

**Keywords:** Stroke, Prosody, Reverse-correlation, Internal noise, Perception, Auditory system, Cognitive neuroscience

## Abstract

After a right hemisphere stroke, more than half of the patients are impaired in their capacity to produce or comprehend speech prosody. Yet, and despite its social-cognitive consequences for patients, aprosodia following stroke has received scant attention. In this report, we introduce a novel, simple psychophysical procedure which, by combining systematic digital manipulations of speech stimuli and reverse-correlation analysis, allows estimating the internal sensory representations that subtend how individual patients perceive speech prosody, and the level of internal noise that govern behavioral variability in how patients apply these representations. Tested on a sample of N = 22 right-hemisphere stroke survivors and N = 21 age-matched controls, the representation + noise model provides a promising alternative to the clinical gold standard for evaluating aprosodia (MEC): both parameters strongly associate with receptive, and not expressive, aprosodia measured by MEC within the patient group; they have better sensitivity than MEC for separating high-functioning patients from controls; and have good specificity with respect to non-prosody-related impairments of auditory attention and processing. Taken together, individual differences in either internal representation, internal noise, or both, paint a potent portrait of the variety of sensory/cognitive mechanisms that can explain impairments of prosody processing after stroke.

## Introduction

After a right hemisphere stroke, more than half of the patients present a communication disorder such as aprosodia, the impossibility to produce or comprehend speech prosody—or the “melody” of speech^1–5^. Despite the social-cognitive implications for patients of not being able to process e.g. linguistic or emotional prosody^6^, aprosodia following stroke has received scant attention.

First, the existing assessment tools for impairments of prosodic processing are found to be lacking in several aspects. The gold standard in the French language, the “Montréal Evaluation de la Communication” (MEC)^7^ consists of a combination of listening and production tests which exhibit good inter-rater reliability but are suspected of limited sensitivity, failing to capture nuanced deficits in language processing in e.g. ecological situations^8^. More generally, traditional pre-post assessments with listening batteries (ex. the 12-items of the MEC prosody task) suffer from test–retest effects, where participants might remember their responses, leading to learning effects. Additionally, assessments based on prosody production typically involve manual scoring by clinicians, which may generate issues of inter-rater variability and limits the potential for monitoring patients remotely. Finally, existing tools typically provide a binary score indicating the presence or absence of a pathology, but do not allow for an in-depth understanding of the mechanisms that explain why a specific error may occur.

Besides lacking sensitive assessment tools, the field is also lacking in its understanding the exact sensory/cognitive mechanisms that subtend aprosodia^4^. On the one hand, a wealth of cognitive neuroscience research has linked linguistic and/or emotional prosody perception with a dominantly-right temporo-frontal network^9^—although it should be noted that recent research has also implicated a wider variety of cortical and subcortical networks^10^. One prominent explanation for such a specialization proposes that the bilateral auditory cortices differ in their temporal and spectral resolution, with left auditory regions responding preferably to fast changes in the type of spectral cues implicated in phonetic discrimination, and right auditory regions to slow variations of pitch as seen in speech prosody and music^11^. On the other hand, clinical patient data has also linked right hemisphere damage due to stroke with a wide multitude of cognitive-communication deficits, which not only include aprosodia, but also impairments of the interpersonal communication such as inappropriate pragmatics and humour^1^, as well as domain-general deficits in attention, memory and executive function^5^. It therefore remains poorly understood whether impairments of prosody perception result from specific damage in regions involved in speech representations, or in more generic mechanisms^4^. Lacking a mechanistic understanding of why patients perform poorly on such tasks deprives health practitioners of practical therapeutic targets for their subsequent rehabilitation.

When studying the neural mechanisms that relate physical stimuli to perception, the modern field of psychophysics has largely moved from simply measuring sensory thresholds and psychometric functions, and now provides a toolbox of techniques to measure and fit multi-staged models able to simulate participant behaviour^12^. Notably for the example of speech prosody, the psychophysical technique of reverse-correlation (or “classification images”)^13^ allows estimating, at the individual level, not only what sensory representations subtend the normal or abnormal perception of e.g. interrogative prosody^14^, but also “internal noise” parameters that capture aspects of behavioral variability that are of potential neurological relevance^15,16^.

While the representation + noise model has a rich history in healthy participants, with or without peripheral hearing impairment^17,18^, its use in participants with neurological or developmental disorders has received relatively little attention^19–21^. Here we show on a sample of N = 22 right-hemisphere brain stroke survivors that such simple procedures promise to enrich the current clinical toolbox with more sensitive and informative markers of receptive aprosodia. While the same tool can be applied to study a variety of prosodic functions, incl. social or emotional, in this study we specifically target the perception of linguistic prosody, defined as the acoustic variations of suprasegmental cues such as tone, amplitude and speech rate that support language analysis beyond the phonetic level, incl. syntax, semantics, and discourse structure^22^—for instance shaping sentences into questions or statements with rising or falling intonations^23^. Using reverse correlation, we show that it is possible to estimate not only the internal representations that subtend how individual patients specifically perceive interrogative prosody, but also a quantitative measure of the consistency with which patients apply these representations in perceptual tasks, and that these two parameters have potential to surpass both the sensitivity and diagnostic richness of existing tools.

## Materials and methods

### Participants

N = 22 brain stroke survivors (male: 17; M = 57 yo, SD = 12.43), and N = 21 age-matched controls (male: 13; M = 58 yo, SD = 13.34) took part in the study. There was no significant sex distribution difference between groups (Chi-square test, p = 0.368), and no significant age difference (Mann–Whitney, p = 0.970).

All patients were in- or out-patients of the Physical Medicine & Rehabilitation Department, APHP Pitié-Salpêtrière Hospital in Paris, France, undergoing speech therapy for different deficits post-stroke like swallowing difficulties, neuro-visual impairments, attentional impairments, neglect, dysphasia etc. Patients included in the study (Table 1) had a history of supratentorial right-hemisphere ischemic stroke, corroborated by clinical assessments NIH stroke scale (NIHSS; M = 10.8) and brain MRI, and dating less than 1y (Median = 4 months) at the time of inclusion; were first-language French speakers; and had no disorders of wakefulness/consciousness, dementia, severe dysarthria, psychiatric antecedents (> 2 months in-patient) or major visual or auditory impairment (> 40 dB HL). Patients with language comprehension deficits -aphasia- (score < 10/15 on the BDAE instruction-following task) were excluded from the study.

**Table 1:** Patients and control demographics and clinical characteristics.

		Controls	Patients
n		21	22
Sex, n (%)	f	8 (38.1%)	5 (22.7%)
	m	13 (61.9%)	17 (77.3%)
Age, median [min, Q1, Q3, max]		58 yo [27,52,64,82]	60.5 yo [28,52.2,63,74]
Month after stroke, median [min, Q1, Q3, max]			4 mo [0,1,5,17]
Stroke type, n (%)	HEM		3 (33.3%)
	ISCH		6 (66.7%)
NIH stroke scale (NIHSS), median [min, Q1, Q3, max]			Available: N = 11(50%) 10 [2,5.5,16,20]
MEC Prosody Comprehension item, median [min, Q1,Q3, max]			Available: N = 22 (100%) 9 [0,8,11,12]
MEC Prosody Repetition item, median [min, Q1, Q3, max]			Available: N = 22 (100%) 11 [7,10,12,12]
MEC Total, median [min, Q1, Q3, max]			Available: N = 22 (100%) 21 [9,18.2,22.8,24]
BDAE command execution item, median [min, Q1, Q3, max]			Available: N = 22(100%) 14 [5,14,15,19]
Audiogram left-ear, median dBHL at 1000 Hz [min, Q1, Q3, max]		0 dBHL [0,0,15,35]	Available: N = 7(31%) 20 dBHL [10,15,30,60]
Audiogram right-ear, median dBHL at 1000 Hz [min, Q1, Q3, max]		5 dBHL [0,0,20,30]	Available: N = 7(31%) 15 dBHL [5,7.5,37.5,45]
Vocal audiogram, median% detection at 40 dB [min, Q1, Q3, max]			Available: N = 13(59%) 99. % [85,94,100,100]
LAMA Sustained auditory attention score accuracy, median [min, Q1, Q3, max]			Available: N = 12(54%) 30 [29,29.8,30,30]
LAMA Sustained auditory attention reaction time (sec), median [min, Q1, Q3, max]			Available: N = 12(54%) 92.5 [63,85.8,137,192]
MBEA (Montreal Battery of Evaluation of Amusia), median [min, Q1, Q3, max]			Available: N = 13(59%) 60 [48,57,71,85]
AIRTAC2 (Auditory discrimination), median [min, Q1, Q3, max]			Available: N = 13(59%) 44 [36,42,47,48]
HADS (depression + anxiety), median [min, Q1, Q3, max]			Available: N = 13(59%) 18.5 [7,11.2,24.8,35]

N = 22 right-hemisphere stroke survivors and N = 22 age-matched controls took part in the study.

MEC, Montréal Evaluation de la Communication; BDAE, Boston Diagnostic Aphasia Examination.

In addition, we recruited a group of N = 21 controls matched in age, sex and degree of hearing loss. Seven of these control participants were recruited via the INSEAD-Sorbonne Université Center for Behavioral Science, Paris, France, and took part in the experiment in a laboratory setting. The remaining 15 were recruited among the FEMTO-ST participant pool, and took part in an online version of the same procedure. Among these 15 online participants, we concluded that one participant was not sufficiently engaged in the task, statistical results conducted with the full control group (including this outlier) are qualitatively similar to our main text conclusions, and presented in Supplementary Text 2.

### Clinical assessment

Two subtests of the French version of the “Montréal Evaluation de la Communication” (MEC) protocol^7^ were administered to the patients to assess their linguistic prosody capacities (comprehension and repetition). The linguistic prosody comprehension subtest evaluated the ability to identify linguistic intonation patterns. This subtest consists of four semantically neutral simple sentences and each one is presented to the patient with three different intonations, for a total of 12 items. After listening to a sentence, the patient is asked to select the correct intonation among the three different written options (interrogative, imperative or affirmative). The linguistic prosody repetition subtest examines the ability to verbally reproduce linguistic intonations. It is formed of the same four sentences as the comprehension task. The previously recorded stimuli are presented in random order. The patient is asked to repeat each sentence with the same intonation. The maximum score is 12 for both subtests.

In order to exclude patients with a significant hearing impairment from the study, patients were assessed using Lafon's cochlear lists of monosyllabic words (List 2 and List 3)^24^. These were calibrated at an intensity of 40 decibels (dB) and played through headphones. Only patients who scored 80% or more on both lists were included. In addition, the Boston Diagnostic Aphasia Examination (BDAE) command execution subtest^25^ was used to exclude patients with comprehension disorders. Only patients with a score of 12/15 or higher were included. Some patients underwent MMSE (Mini-Mental State Examination) or MoCA (Montreal Cognitive Assessment) evaluations as part of their clinical follow-up, but these assessments were conducted at different times, making it difficult to perform direct comparisons. It's important to note that the Boston Diagnostic Aphasia Examination (BDAE) command execution subtest^25^ was used to exclude patients with comprehension disorders. Only patients with a score of 12/15 or higher were included. And none of these patients suffered from aphasia, as it was an exclusion criterion for our study.

To assess possible mood disorders, the Hospital Anxiety and Depression Scale (HADS) self-questionnaire^26^ was administered to patients to assess their current level of anxiety and depression. It contains 7 questions for the anxiety part and 7 questions for the depression part, with a separate score for each. A score of 11 or more for each part indicates a possible anxiety and/or depression state.

To assess auditory attention, a subset of patients also underwent the sustained auditory attention subtest of the “Logiciel d’Attention en Modalité Auditive'' (LAMA)^27^. The assessment and rehabilitation software “Aide Informatisée pour la Rééducation des Troubles Auditifs Centraux '' (Airtac2)^28^ was used to assess central auditory processing. Intensity discrimination and duration discrimination of non-verbal sounds were proposed to compare central auditory processing abilities with the results of the Reverse Correlation task. Finally, the Montreal Battery of Evaluation of Amusia (MBEA)^29^ was selected to assess the music perception abilities of a subset of patients. Since the disorder of music perception (amusia) is primarily a disorder of pitch perception^30^, the three tasks in the melodic organization part (scale test, contour test, interval test) were selected (See Table 1 for details).

### Procedure

We recorded a 426-ms utterance of the French word “vraiment” (“really”), and generated prosodic variations by dividing it into six segments of 71 ms and randomly manipulating the pitch of each breakpoint independently using a normal distribution (SD = 70 cents; clipped at ± 2.2 SD), hereafter referred to as “stimulus noise”. These values were linearly interpolated between time points and fed to an open-source pitch-shifting toolbox (CLEESE, Python language, v1.0, available at https://github.com/neuro-team-femto/cleese) developed for this purpose^31^. We then presented patients with 150 successive pairs of such manipulated utterances (*really/really?*) asking them to judge which, within each pair, sounded most interrogative (examples of sound stimuli are available in the code repository shared with the article—see *Code Availability*). The sequence was divided into 3 blocks of 50 pairs. Without the participant’s knowing, the first and last block of each sequence contained identical pairs of sounds (a procedure called double-pas^15,32^, allowing us to examine response variability), but all other sounds in the sequence were otherwise distinct (in more details, N = 9/22 patients and N = 7/21 controls had only 25 repeated trials among block 2 and 3, while the other N = 13/22 patients and N = 14/21 controls had a complete repetition of the 50 trials in blocks 2 and 3; there was no statistical difference between the levels of internal noise measured with these two setups (patients: Mann–Whitney p = 0.24; controls: p = 0.13). N = 9/22 patients were additionally tested 4 repeated (one week apart), but we have only retained the first session and did not include these extra data points in the statistical analysis. Sounds were delivered using closed headphones (Beyerdynamics DT770) presented the stimuli dichotically (same signal in both ears) at an identical comfortable sound level (~ 70 dB SPL) to all patients and healthy subjects. The inter-stimulus interval in each pair was 500 ms, and the interval between successive pairs was 1 s. The procedure took about 15 min to complete.

### Reverse-correlation analysis

**Figure 1:**
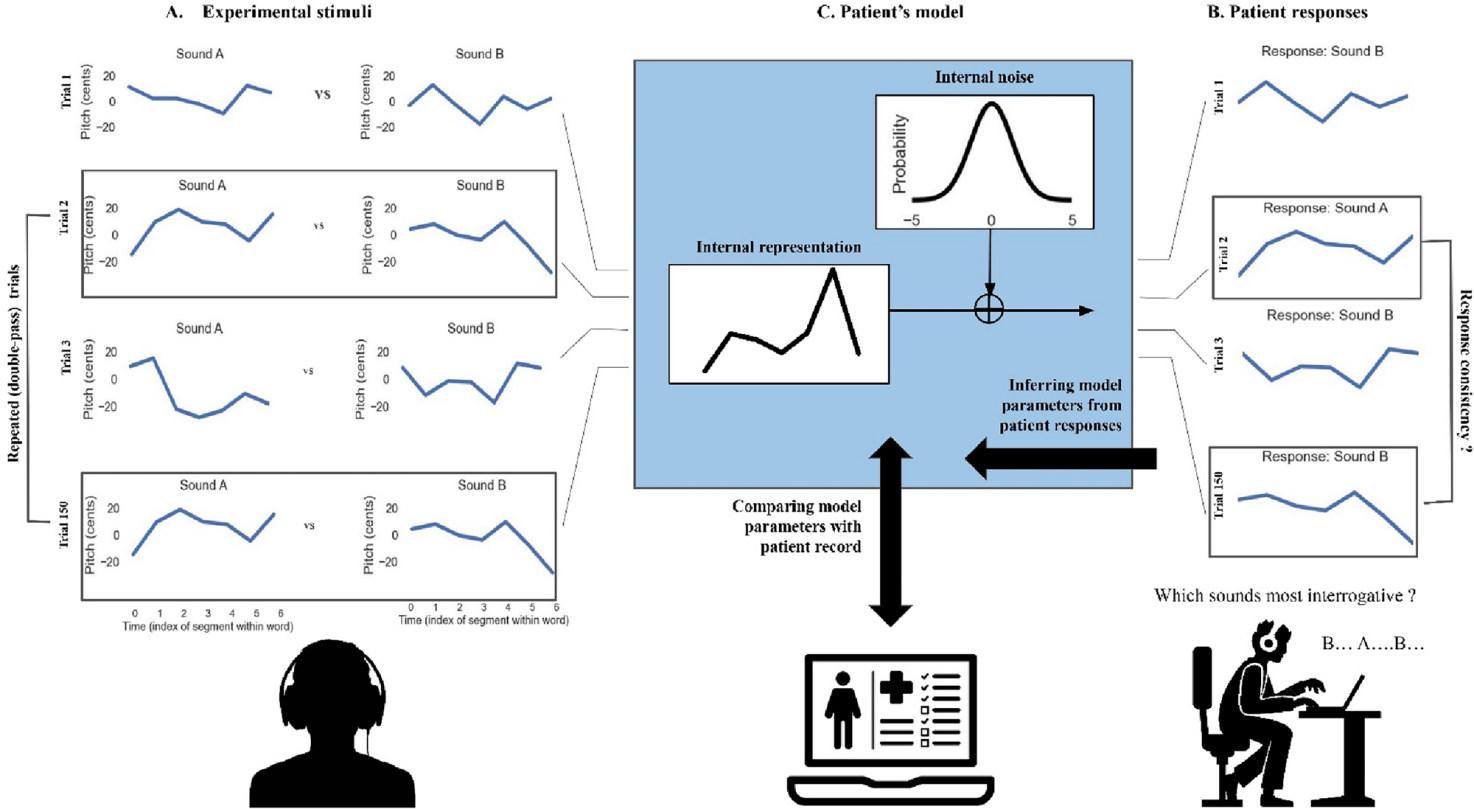
The representation + noise model. Patients were presented with 150 successive trials consisting of pairs of manipulated prosodies (**A**) and asked to judge, within each pair, which sounded most interrogative (**B**). Patient responses in each trial were fitted with a 2-stage psychophysical model (**C**), consisting, first, of a prosodic template (or “internal representation”) to which sound stimuli are compared and, second, of a level of “internal noise” which controls how consistently this representation is applied to incoming stimuli. See main text for details about the model-fitting procedure. In this work, we estimate the two model parameters (representation and noise) for each patient individually and compare them with patient records to test their value as markers of receptive aprosodia.

For each participant's response data, we fitted a 2-stage psychophysical model consisting, first, of a prosodic template (or “internal representation”) to which sound stimuli are compared and, second, of a level of “internal noise” which controls how consistently this representation is applied to incoming stimuli (Fig. 1).

Participants' internal representations (a time × pitch representation of an ideally interrogative pitch contour) were computed using the classification image technique^13^ to differentiate between interrogative and non-interrogative pitch contours. Specifically, we subtracted the average pitch contour of non-interrogative classifications from that of interrogative classifications. To normalize this resultant representation, we divide it by the root mean square of its values—this method involves squaring each value of the representation, averaging these squared numbers, and then taking the square root of this average to scale the representation accordingly. For each patient, we then quantified how similar their internal representation is to the average representation in the control group, by computing the mean squared error between the two representations, and used this “representation typicality” as a parameter to correlate with clinical measures. Representations for controls were computed using the same procedure, using only the first 150 trials of each session in order to match the number of trials seen by patients.

Participants’ internal noise (expressed in units of the standard deviation of stimulus noise) was inferred from response consistency and response bias across the repeated double-pass trials, using the simulation procedure of Neri^15^. In short, we computed an idealized participant model responding to repeated stimuli pairs of various sensory evidence, perturbed its response with additive gaussian noise (“internal noise”), and estimated the probability for that model to give the same response for identical trials (i.e. response consistency) and the probability of giving the first response option (i.e. response bias), for different standard deviations of that internal noise. For each participant, we then inverted that model and obtained the value of internal noise (by exhaustive search between 0 and + 5 std) that minimized the error between the observed and predicted values for that participant’s consistency and bias. As in previous studies^15^, we estimated internal noise conservatively between [0; + 5 std] in order to avoid unreliable estimates at large values, a known problem with double-pass procedures (see Appendix A). Internal noise values in the upper side of that range (e.g. illustrated in Fig. 3 between 4.8 and 5) may either correspond to true internal noise values, or to larger values for which we could not provide an exact estimate.

Both of these analyses (internal representations and internal noise) were conducted using an open-source Python toolbox built for this purpose (PALIN v1.0, Python language, v1.5, available at https://github.com/neuro-team-femto/palin).

### Statistical analysis

Group comparisons: because distributions of representation typicality and internal noise scores between patients and controls were non-normal, we compared population means using non-parametric (Mann–Whitney) independent sample t-tests.

Correlation with clinical measures: linear associations between representation typicality and internal noise, and clinical assessments (MEC, Prosody Comprehension, Prosody Repetition, Airtac2) met the homoskedasticity assumption and were therefore estimated using ordinary least-square regressions without robust (HC) norms, as these are considered to increase false positive rates when testing small samples. In addition, because regression residuals were occasionally non-normal, we estimated statistical significance using bootstrapped confidence intervals^33^. The analysis was implemented with the pymer.lm package^34^ v4 0.8.2.

### Ethics statement

The study was approved by *Comité de Protection des Personnes* CPP Ile-De-France V (ProsAVC, Decision of 22/07/2020). All methods in this study were carried out in accordance with the relevant guidelines and regulations, and all data in this study were obtained with informed consent from all subjects and/or their legal guardian(s).

## Results

**Figure 2:**
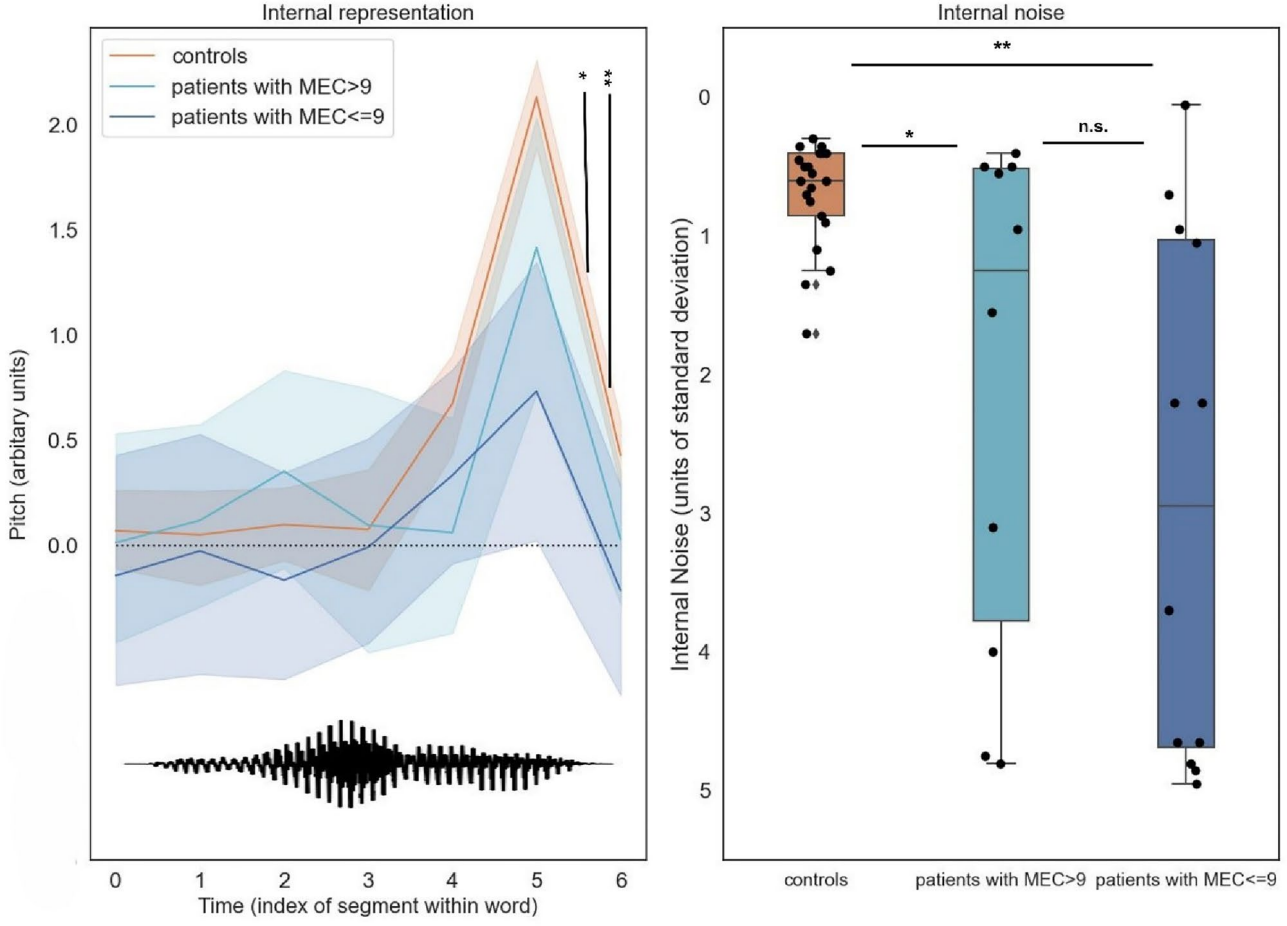
Patient parameters (internal representations and internal noise) estimated by reverse-correlation separate controls from patients above and below the pathological cut-off on the MEC prosody comprehension scale (9/12). Left: Internal representations of interrogative prosody computed from control group responses exhibited a typical final-rise contour, with a marked increase of pitch at the end of the second syllable. In contrast, patients’ internal representations had both lower amplitude and more variable shape across individuals. The bottom waveform illustrates the shape of the base sound used to generate stimuli (a male-recording of the word vraiment/really). Right: control participants were able to apply these representations remarkably consistently across trials, with internal noise values < 1 standard deviations of stimulus noise. In contrast, patients’ internal noise levels were larger and more variable, and scaled with prosodic difficulties measured by MEC.

**Figure 3:**
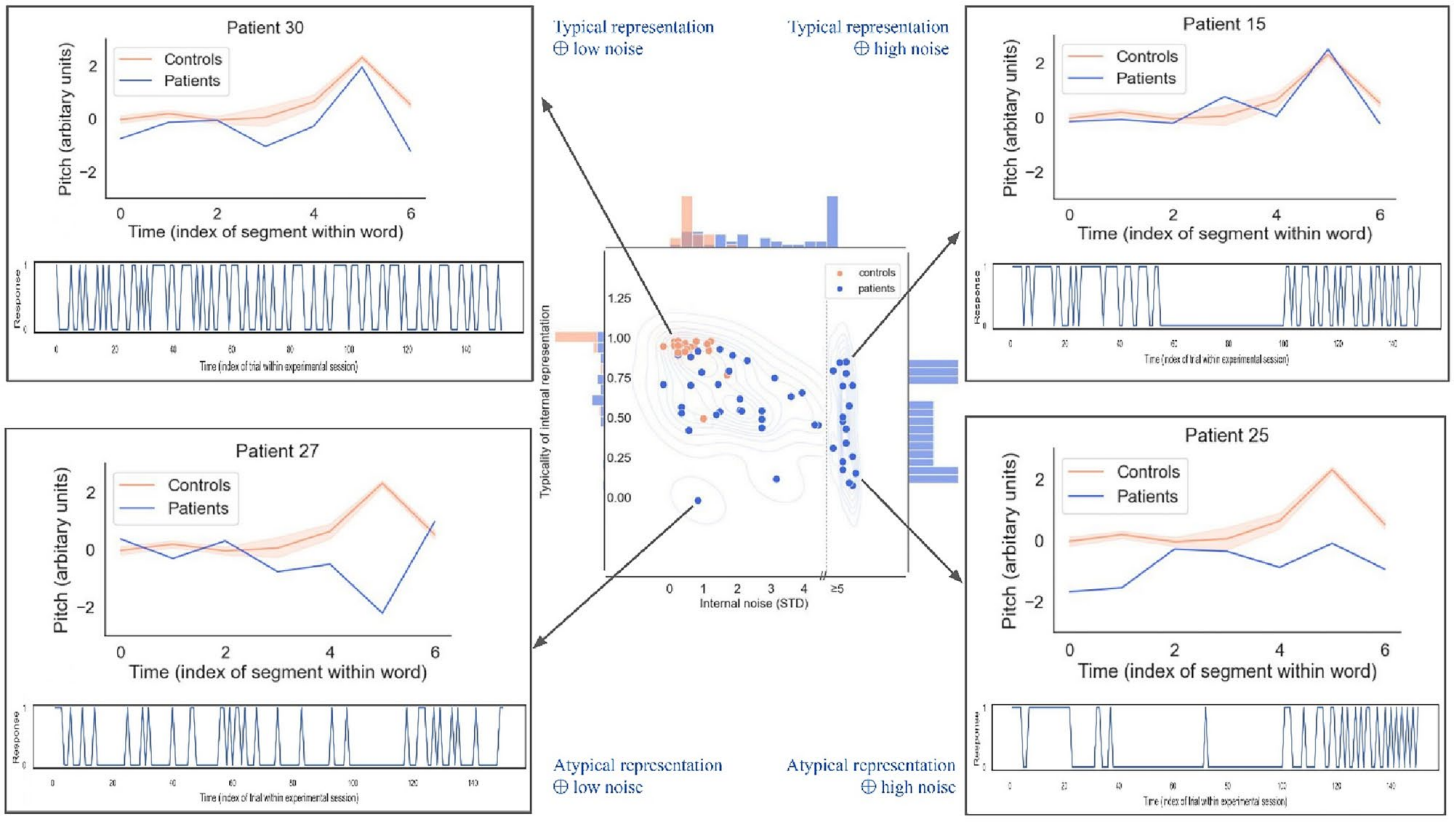
The representation + noise model captures a rich diversity of sensory/cognitive mechanisms underlying impairments of prosody processing after stroke. Center: Distribution of representation typicality and internal noise for controls and patients (considering all 4 sessions), overlaid with by kernel density estimate. Histograms on the marginal axes show univariate distributions for each variable in the patient group. Corners: Corner boxes show internal representations (top) and behavioral series of responses (bottom) for 4 illustrative patients. Patients in top corners have internal representations (blue) that are similar to controls (orange), but vary in amounts of internal noise (e.g. showing excessive response perseveration; top-right). Patients in bottom corners have atypical representations (blue), but some nevertheless retain healthy levels of internal noise (e.g., being normally consistent in wrongly expecting question phrases to decrease rather than increase in pitch; bottom-left). The estimation of internal noise was limited to the range [0; + 5std]; data points in the upper side of that range may either correspond to true internal noise values, or to larger values for which we could not provide an exact estimate, as illustrated here with a dotted line in the central panel (see Appendix for details).

Both measures extracted from the reverse-correlation procedure allowed separating patients from controls: internal representations of interrogative prosody computed from control group responses exhibited a typical final-rise contour^14^, with a marked increase of pitch at the end of the second syllable (Fig. 2-left), and control participants were able to apply these representations remarkably consistently across trials, with internal noise values M = 0.7 (SD = 0.37) in the range of those typically observed for lower-level auditory and visual tasks^15^ (Fig. 2-right). In contrast, patients’ internal representations had both lower amplitude (indicating less discriminative power) and more variable shape across individuals (Fig. 2-left; see also Fig. 3), and were applied with higher levels of internal noise (M = 2.54, SD = 1.90; Fig. 2-right). The two groups differed statistically for both representation typicality: M = 0.27 [0.16; 0.39], Mann–Whitney’s U(− 0.82) = 420, p < 0.001; and internal noise: M = − 1.84 [− 2.61; − 1.07], U(0.59) = 95.00, p = 0.001.

Within the patient group, internal noise values (and, to a lower extent, representation typicality) were statistically associated with scores of the current gold standard for assessing deficits of prosody perception (MEC), demonstrating good concurrent validity. First, larger internal noise values were associated with lower (more severe) scores on the MEC prosody comprehension scale: noise: R^2^ = 0.189, β = − 0.303 [− 0.596; − 0.010], t(20) =  − 2.158, p = 0.043. Representation typicality also improved with better scores, albeit non-statistically (R^2^ = 0.100, β =  + 0.03 [− 0.012; + 0.071], t(20) = 1.49, p = 0.15). Second, both measures had also good symptom specificity, as strikingly neither correlated with the MEC score for prosody repetition (representation: R^2^ = 0.002, t(20) =− 0.219, p = 0.82, noise: R^2^ = 0.041, t(20) =  − 0.92, p = 0.365), while both MEC scores were themselves positively correlated (r = 0.53).

An oft-quoted limitation of the MEC instrument is its poor sensitivity, with patients above the pathological cut-off on the MEC prosody comprehension scale (9/12) still complaining of communication difficulties^8^. Interestingly, our measures allowed clear separation of this group of MEC-negative patients (i.e. patients with MEC > 9) (N = 12/22) and controls (N = 21), both in terms of typicality of representation (M = 0.18 [0.06; 0.32], U (− 0.74) = 219.0, p = 0.001) and internal noise (M = − 1.54 [− 2.62; − 0.53], U(0.48) = 66, p = 0.026).

Finally, to examine the convergent validity and specificity of internal representation and internal noise measures, we investigated whether they were statistically associated with other constructs linked to central deficits common in stroke rehabilitation. Expectedly, both measures were associated with difficulties discriminating tone intensity and tone duration, as measured by AIRTAC2 (representation: R^2^: 0.49, β =  + 0.040 [0.013; 0.068], t(11) = 3.27, p = 0.007; noise: R^2^: 0.33, β = − 0.28 [− 0.54; − 0.020] t(11) =  − 2.36, p = 0.037). However, they were not associated with the patient’s capacity to detect rare auditory targets among distractors, as measured by LAMA (representation: R^2^: 0.130, t(10) = 1.22, p = 0.25; noise: R^2^: 0.136, t(10) =  − 0.125, p = 0.23); or with the patient’s capacity to process musical melodies, as measured by MBEA scale and melody items (representation: R^2^: 0.050, t(11) = 0.765, p = 0.46; noise: R^2^: 0.00, t(11) = 0.027, p = 0.98). Regarding music ability in particular, MBEA was assessed in N = 13 (59%) of our patients, the majority of which N = 8 (62%) were found impaired for melody/pitch processing with scores below the pathological cut-off score of 65/90. Out of the 8 patients who scored with melody amusia, 6 (75%) had representations that visually departed from controls. Comparatively, 3 out 5 (60%) of the patients without amusia had normal representations (Fig. S2).

Finally, internal noise (but not representation typicality) was found related to patients’ level of anxiety and depression, as measured by HADS (noise R^2^ = 0.249; β = 0.108 [0.021; 0.196], t(20) = 2.57, p = 0.018; representation R^2^ = 0.089, t(20) =  − 1.39, p = 0.178).

## Discussion

In this report, we introduced a novel, simple psychophysical procedure which, by combining systematic digital manipulations of speech stimuli and reverse-correlation analysis, allows estimating the internal sensory representations that subtend how individual patients perceive speech prosody, as well as the level of internal noise that govern behavioral variability in how patients apply these representations in prosodic perceptual tasks.

Tested on a sample of N = 22 right-hemisphere stroke survivors, our two proposed parameters of representation typicality and internal noise provide a promising alternative to the clinical gold standard for evaluating impairments of prosody processing (MEC). First, internal noise (and, to a lesser extent, internal representations) strongly associate with receptive aprosodia, and not expressive aprosodia, measured respectively by MEC recognition and repetition scores within the patient group. Second, internal representations (and, to a lesser extent, internal noise) have better sensitivity than MEC for separating high-functioning patients from controls. Finally, both measures appear to have relatively good specificity with respect to non-prosody-related impairments of auditory attention and auditory processing, although internal noise was also found associated with mood disorders which, in our sample, were also predictors of MEC scores.

The fact that abnormal internal representations in our patient sample correlate with performance in prosody recognition but not repetition prompts the question whether impairments in perceptive representations are dissociated from impairments in mapping process between these representations and the corresponding phonatory and articulatory commands involved in their production. On the one hand, the MEC “repetition task”, which consists of hearing a target expression produced by the therapist and subsequently reproducing it vocally, does not necessarily involve perceptual representations associated with the recognition of the expression as being e.g. interrogative. It could in principle result from the direct sensorimotor mapping of the auditory characteristics of the stimuli to the corresponding pattern of phonatory (in the case of pitch) and articulatory (in the case e.g. of phonemes or timbre) motor commands. It follows that low recognition scores could in principle be associated with good repetition scores (which indeed we’re seeing in a good share of our patients, see upper-left quadrant in Supplementary Fig. S1). This pattern of results is consistent e.g. with literature showing imitation of vocal gestures (such as smiling) without their simultaneous recognition^35^. On the other hand, a wealth of research has documented strong links between action and perception in imitation tasks, and notably established that imitation or action simulation has a causal role in facilitating recognition^36^. For instance, blocking the imitation of a facial expression has detrimental behavioural^37^ and neurophysiological effects^38^ on their simultaneous recognition. In that sense, it could be expected that patients with low repetition scores would also show low recognition scores (which is again consistent with the low number of data point in the bottom-right quadrant of Fig. S1). To further investigate these links, it would be interesting to collect additional data in which we specifically ask patients to vocalize interrogative prosodies (without providing any auditory examples), and examine the correspondence between their recognition kernel and their produced pitch profiles.

The fact that a majority of patients tested with abnormal melodic processing abilities (MBEA < 65) also had impaired prosodic representations (although the opposite was not true, see Fig. S2) brings questions about the sensory/cognitive resources shared between speech and music processing. First, this pattern of results suggests that melodic processing and prosodic representations are at least partially subtended by common domain-generic mechanisms, plausibly linked to pitch contour processing. Such a mechanism would be consistent with previous research showing impairments of amusic patients in distinguishing questions from statements^30^, emotional prosody^39^ and discriminating lexical tones in tone language^40^. Second, it remains that intact pitch/melodic processing is not sufficient to maintain intact prosodic representations (which are impaired in 2/5 of MBEA-positive patients; Fig. S2-top). For this latter subset of patients, impaired representations could result from higher-level lexical or semantic impairments such as difficulties integrating pitch and phonemic information (e.g. failing to associate increasing pitch with the second phoneme of the word “vrai-ment”), or from an impaired semantic representation of what is a question and how it should sound like (e.g. some patients may be consciously expecting that questions are associated with an initial pitch rise). This would be consistent with previous research showing stronger evidence of shared processes between speech and music at earlier and subcortical levels^41^ than e.g. in processes of lexical or semantic verification^42^. Further work could look at these possibilities by e.g. testing patients with monosyllabic words (aah?) or a non-semantic task in which patients have to identify which of two alternatives sounds more like a sound target (which only incidentally sounds like a question).

More generally, while our study includes right-hemisphere damage (RHD) patients based on a wealth of clinical literature associating stroke-related RHD with receptive aprosodia^1,4,5,43^, our results are only correlational and merely observing changes in internal representation and internal noise in patients with right-hemisphere lesions does not necessarily mean these effects are caused by the right hemisphere damage. Without more direct evidence, one can only speculate about the possible neurological bases for these two types of abnormalities. In terms of representations, one might imagine the involvement of sensory areas, possibly lateralized and specialized for e.g. vocal sounds and/or the slow-varying spectral changes that are characteristics of prosodic pitch contours (e.g. right STG^44^). Regarding internal noise, we may be looking at more diffuse causes, possibly involving frontal areas, and possibly less lateralized^4^. To further look into these questions, future studies could examine possible dissociations with other types of lesions (typically, are left hemisphere stroke patient similarly impaired in representations and/or noise) or use lesion-symptom mapping approaches within a RHD group to link both types of impairment to possibly more specific right areas^46^.

In this study, we have focused on a specific type of linguistic prosodic function, namely the marking of interrogation by a final pitch rise. Our focus on interrogative prosody in the present task should by no means be taken as a proposal that it constitutes the optimal test providing most coverage of stroke-related prosodic impairments, but rather as a proof of concept. The reverse-correlation paradigm lends itself ideally to investigate a wide range of other tasks, such as pitch contour representations in other types of linguistic prosody (e.g. imperative sentences to complement the items available in MEC, or prosodic cues to word boundaries^47^), emotional or social prosody (e.g. dominance and trustworthiness^14^); but also other acoustic domains that pitch, such as loudness and speech rate^48^ or timbre/phonological cues as used e.g. in phoneme classification^49^. Because of its versatility, reverse correlation appears as a promising way to evaluate prosodic perception mechanisms mechanistically across such a wide range of tasks and cues.

In sum, the representation + noise model paints a simple yet potent portrait of the variety of sensory/cognitive mechanisms that can explain impairments of prosody processing after stroke: patients may differ from controls by having altered representations but a healthy level of internal noise (e.g., being *normally* consistent in *wrongly* expecting e.g. question phrases to decrease rather than increase in pitch—Fig. 3-left); by having normal representations but abnormal levels of internal noise (e.g. showing excessive response perseveration and suboptimal executive control on top of otherwise *normal* sensory processing—Fig. 3-right); or both.

By separating these different profiles of pathology, it is our hope that the representation + noise model will provide more effective and individualized therapeutic targets for rehabilitation of individuals with impaired speech prosody perception than existing measures^50^. Our data indicate that deficits in prosody perception can stem both from attentional/executive or representational problems, underscoring our approach’s utility in revealing the underlying mechanisms behind individual patients' comprehension difficulties. Importantly, not all patients with attentional challenges will exhibit aprosodia^1^, which positions our method as a complement to, rather than a replacement for, traditional attention assessments by pinpointing the specific contributors to perceptual difficulties. This effort aims to enrich our understanding and assessment of the complex nature of prosody perception and its deficits. For example, patients with the highest levels of internal noise may benefit from therapies that focus on attentional and executive skills, or from transcranial brain stimulation, which has been found to selectively manipulate internal noise in visual tasks^51^. Similarly, for patients encountering difficulties at the internal representation stage, targeted interventions could emphasize pitch contour discrimination or melody imitation tasks, potentially augmented with visual feedback to bolster the reformation of accurate internal representations of prosodic and musical elements^8,52^. Finally, regarding clinical functionality, while the reverse-correlation procedure is, for now, comparable in duration with the MEC perception tasks (MEC: M = 10–15 min, revcor: M = 15 min), it is also easy to dispense remotely (the current control sample was collected with an online app, https://github.com/neuro-team-femto/revcor), does not require supervision or manual scoring, and can be optimized to even shorter durations using e.g. genetic programming optimizations that continuously adapt the presented stimuli to the patient’s previous responses^53^. With such adaptations, the reverse correlation procedure could be used to evaluate the prognostic value of measuring changing levels of representation typicality and noise longitudinally, along the weekly or even daily course of rehabilitation.

### Supplementary Information

Supplementary Information 1.

Supplementary Information 2.

## Data Availability

The datasets used and/or analyzed during the current study available from the corresponding author on reasonable request.

## Code availability

Experimental procedures, stimuli, code for analyzing the data, as well as a selection of control (but not patient) data are available at https://github.com/neuro-team-femto/revcor_avc_public. The online application for collecting control data is available at https://github.com/neuro-team-femto/revcor. Sound stimuli were generated with the CLEESE toolbox, Python language, v1.0, available at https://github.com/neuro-team-femto/cleese. Kernel and internal noise analysis done with the PALIN toolbox, Python language, v1.0, available at https://github.com/neuro-team-femto/palin.
