# Supplementary material for: A simple psychophysical procedure separates representational and noise components in impairments of speech prosody perception after right-hemisphere stroke
Source: Sci Rep. 2024 Jul 2;14:15194. doi: 10.1038/s41598-024-64295-y (PMC11219855; doi:10.1038/s41598-024-64295-y)
Supplement: Supplementary file 2 — Supplementary Information 2. [file 41598_2024_64295_MOESM2_ESM.docx]

## Appendix

## Supplementary Text 2. Results without rejecting outlier control participant

Although we collected data from N=22 control participants, matching the number of patients, one control participant (#6) had an abnormally high level of internal noise (IN=4.9), which is more than 10 SD > the mean M=0.7. Although this participant did not exhibit any obvious sign of technical problem (all trials completed), their performance metrics were in the most extreme range of the group: their response time to trial blocks 2 and 3, reps. 764ms and 777ms, are in the lowest 30% quantile of all responses; their probability of answering the first response option (p=0.73) is in the top 25% quantile, and their agreement probability in the lowest 25% quantile of all controls, perhaps suggesting that this participant's performance did not reflect cognitive diversity, but rather a low-engagement in the procedure (conducted online for controls 0-14).

For all these reasons, we have opted for excluding this control participant from the main text analysis in comparisons with the patient group, thus reducing our control group to N=21 participants. We added a paragraph discussing the possibility that internal noise measures may be more diverse in the general population than implied by our small group here.

We present here statistical results conducted with the full control group (i.e. including control #6); they are qualitatively similar to our main-text conclusions.

**Descriptive statistics of the control group:**

- **Without participant #6**: N=21 (male: 13; M=58 yo, SD=13.34); no significant sex distribution difference with patient group: p=0.97
- **With participant #6**: N=22 (male: 14;M=58 yo, SD=13.12); no significant sex distribution difference with patient group: p=0.93

**Comparison between patients and controls:**

- **Without participant #6**:

Both measures extracted from the reverse-correlation procedure allowed separating patients from controls: internal representations of interrogative prosody computed from control group responses exhibited a typical final-rise contour[^14^](https://www.zotero.org/google-docs/?ZeW4Kj), with a marked increase of pitch at the end of the second syllable, and control participants were able to apply these representations remarkably consistently across trials, with internal noise values M=0.7 (SD=0.37) in the range of those typically observed for lower-level auditory and visual tasks[^15^](https://www.zotero.org/google-docs/?BGsJdO). In contrast, patients’ internal representations had both lower amplitude (indicating less discriminative power) and more variable shape across individuals , and were applied with higher levels of internal noise (M=2.54, SD=1.89). The two groups differed statistically for both representation typicality: M=0.27 [0.16 ; 0.39], Mann-Whitney's U(-0.82)=420, p<0.001; and internal noise: M= -1.84 [-2.61 ; -1.07], U(0.59)=95.00, p=0.001

- **With participant #6**:

Both measures extracted from the reverse-correlation procedure allowed separating patients from controls: internal representations of interrogative prosody computed from control group responses exhibited a typical final-rise contour[^14^](https://www.zotero.org/google-docs/?c2DtUc), with a marked increase of pitch at the end of the second syllable (Figure 2-left), and control participants were able to apply these representations remarkably consistently across trials, with internal noise values M=0.89 (SD=0.96) in the range of those typically observed for lower-level auditory and visual tasks[^15^](https://www.zotero.org/google-docs/?U0j6uT). In contrast, patients’ internal representations had both lower amplitude (indicating less discriminative power) and more variable shape across individuals, and were applied with higher levels of internal noise (M=2.54, SD=1.89). The two groups differed statistically for both representation typicality: M=0.27 [0.16 ; 0.38], Mann-Whitney's U(-0.80)=436, p<0.001; and internal noise: M= -1.65 [-2.51 ; -.78], U(0.54)=112.50, p=0.002


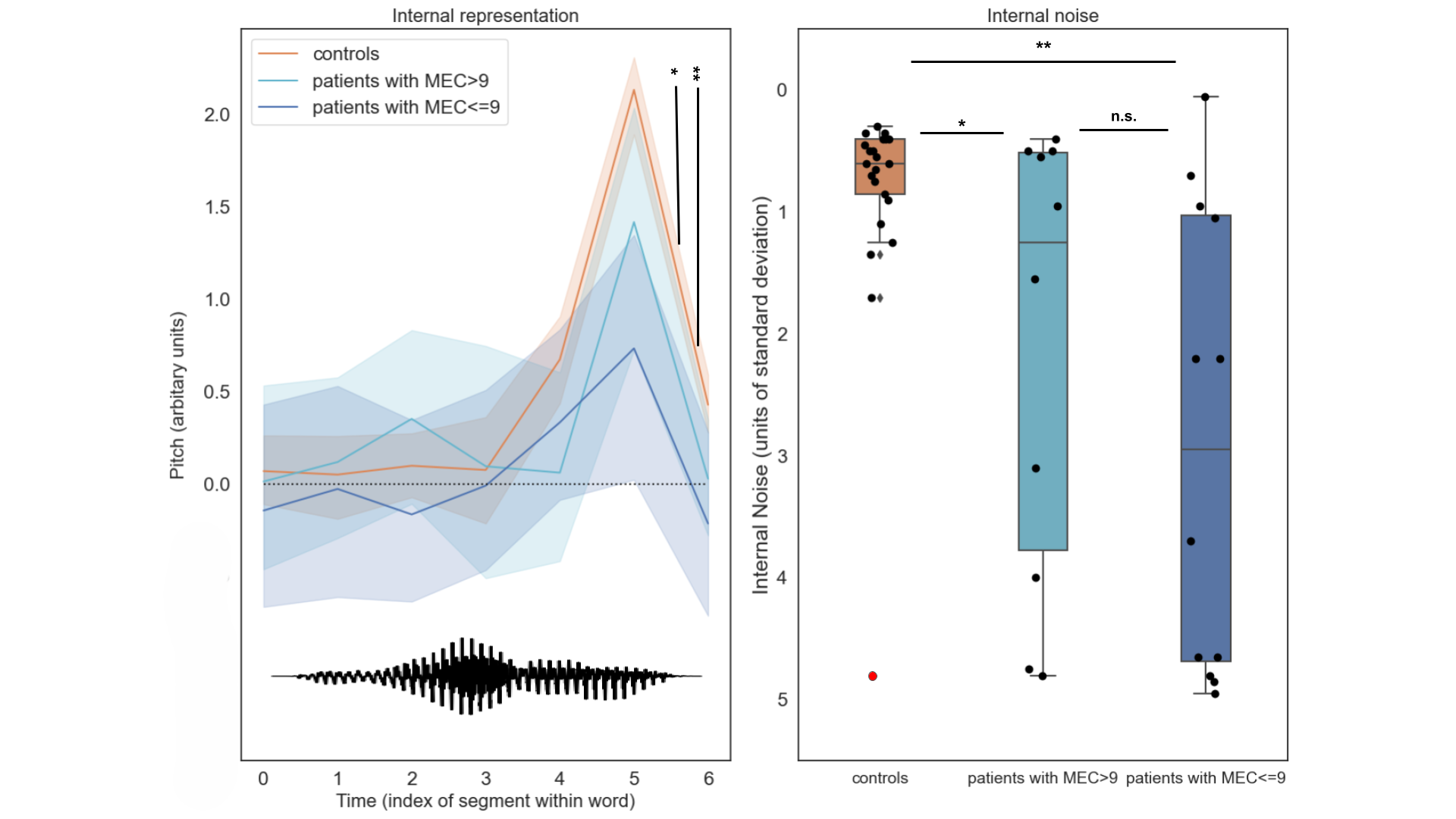


**Figure 2'. Patient parameters (internal representations and internal noise) estimated by reverse-correlation separate controls from patients above and below the pathological cut-off on the MEC prosody comprehension scale (9/12). Left**: Internal representations of interrogative prosody computed from control group responses exhibited a typical final-rise contour, with a marked increase of pitch at the end of the second syllable. In contrast, patients’ internal representations had both lower amplitude and more variable shape across individuals. The bottom waveform illustrates the shape of the base sound used to generate stimuli (a male-recording of the word vraiment/really). **Right**: control participants were able to apply these representations remarkably consistently across trials, with internal noise values < 1 standard deviations of stimulus noise. In contrast, patients’ internal noise levels were larger and more variable, and scaled with prosodic difficulties measured by MEC. The control participant marked in red is participant #6, which was excluded from main text statistical analysis .

**Comparison between controls and MEC-negative patients:**

- **Without participant #6**:

An oft-quoted limitation of the MEC instrument is its poor sensitivity, with patients above the pathological cut-off on the MEC prosody comprehension scale (9/12) still complaining of communication difficulties[^8^](https://www.zotero.org/google-docs/?qHpRwt). Interestingly, our measures allowed clear separation of this group of MEC-negative patients (i.e. patients with MEC>9) (N=12/22) and controls (N=21), both in terms of typicality of representation (M=0.18 [0.06 ; 0.32], U (-0.74)= 219.0, p=.001) and internal noise (M = -1.54 [-2.62 ; -0.53], U(0.48)=66, p=0.026).

- **With participant #6**:

An oft-quoted limitation of the MEC instrument is its poor sensitivity, with patients above the pathological cut-off on the MEC prosody comprehension scale (9/12) still complaining of communication difficulties[^8^](https://www.zotero.org/google-docs/?Q3gUeq). Interestingly, our measures allowed clear separation of this group of MEC-negative patients (i.e. patients with MEC>9) (N=12/22) and controls (N=22), both in terms of typicality of representation (M=0.18 [0.06 ; 0.32], U (-0.70)= 225.0, p=.001) and internal noise (M = -1.31 [-2.44 ; -0.24], U(0.42)=77, p=0.049).

**Figure 3 in the main text including the participant #6**


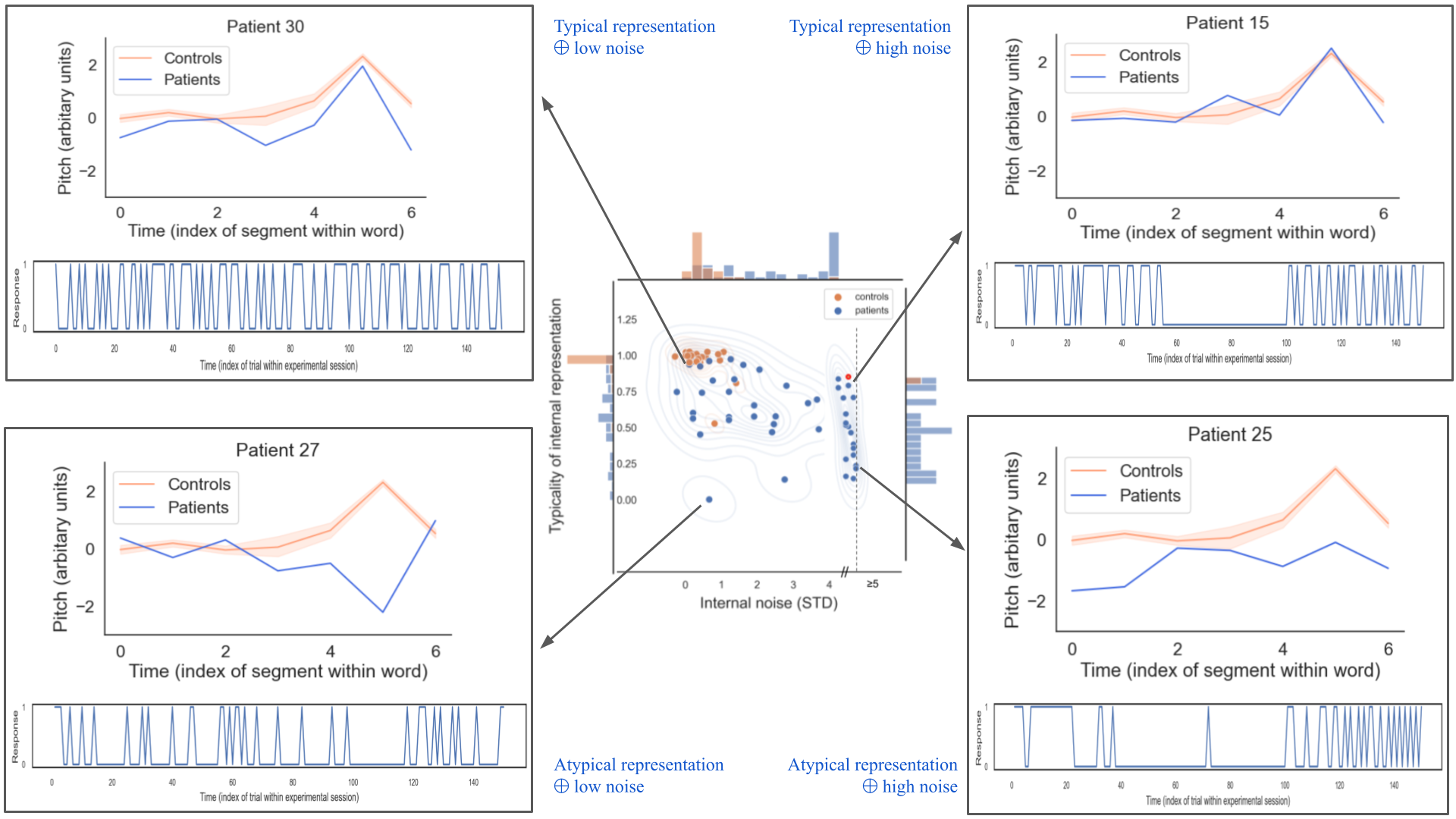


**Figure 3'**. **The representation+noise model captures a rich diversity of sensory/cognitive mechanisms underlying impairments of prosody processing after stroke.** **Center**: Distribution of representation typicality and internal noise for controls and patients (considering all 4 sessions), overlaid with by kernel density estimate. Histograms on the marginal axes show univariate distributions for each variable in the patient group. The control participant marked in red is participant #6, which was excluded from main text statistical analysis (see Supplementary Text 2). **Corners**: Corner boxes show internal representations (top) and behavioral series of responses (bottom) for 4 illustrative patients. Patients in top corners have internal representations (blue) that are similar to controls (orange), but vary in amounts of internal noise (e.g. showing excessive response perseveration; top-right). Patients in bottom corners have atypical representations (blue), but some nevertheless retain healthy levels of internal noise (e.g., being normally consistent in wrongly expecting question phrases to decrease rather than increase in pitch; bottom-left). The estimation of internal noise was limited to the range [0; + 5std]; data points in the upper side of that range may either correspond to true internal noise values, or to larger values for which we could not provide an exact estimate, as illustrated here with a dotted line in the central panel (see Appendix for details).
